# Effect of AI-assisted software on inter- and intra-observer variability for the X-ray bone age assessment of preschool children

**DOI:** 10.1186/s12887-022-03727-y

**Published:** 2022-11-08

**Authors:** Kai Zhao, Shuai Ma, Zhaonan Sun, Xiang Liu, Ying Zhu, Yufeng Xu, Xiaoying Wang

**Affiliations:** Department of Radiology, Peking University First Hospital, Beijing, China

**Keywords:** Bone age, Pediatric, Radiographs, Artificial intelligence, Variability

## Abstract

**Background:**

With the rapid development of deep learning algorithms and the rapid improvement of computer hardware in the past few years, AI-assisted diagnosis software for bone age has achieved good diagnostic performance. The purpose of this study was to investigate the effect of AI-assisted software on residents’ inter-observer agreement and intra-observer reproducibility for the X-ray bone age assessment of preschool children.

**Methods:**

This prospective study was approved by the Institutional Ethics Committee. Six board-certified residents interpreted 56 bone age radiographs ranging from 3 to 6 years with structured reporting by the modified TW3 method. The images were interpreted on two separate occasions, once with and once without the assistance of AI. After a washout period of 4 weeks, the radiographs were reevaluated by each resident in the same way. The reference bone age was the average bone age results of the three experts. Both TW3-RUS and TW3-Carpal were evaluated. The root mean squared error (RMSE), mean absolute difference (MAD) and bone age accuracy within 0.5 years and 1 year were used as metrics of accuracy. Interobserver agreement and intraobserver reproducibility were evaluated using intraclass correlation coefficients (ICCs).

**Results:**

With the assistance of bone age AI software, the accuracy of residents’ results improved significantly. For interobserver agreement comparison, the ICC results with AI assistance among 6 residents were higher than the results without AI assistance on the two separate occasions. For intraobserver reproducibility comparison, the ICC results with AI assistance were higher than results without AI assistance between the 1st reading and 2nd reading for each resident.

**Conclusions:**

For preschool children X-ray bone age assessment, in addition to improving diagnostic accuracy, bone age AI-assisted software can also increase interobserver agreement and intraobserver reproducibility. AI-assisted software can be an effective diagnostic tool for residents in actual clinical settings.

## Background

X-ray bone age assessment (BAA) in children and adolescents is a very important tool for pediatricians in the diagnosis of endocrine and metabolic diseases related to growth and development [1]. It is well known that the Greulich-Pyle (GP) and the Tanner-Whitehouse 3 (TW3) methods are the most commonly used clinical approaches for BAA [2, 3]. The GP method is an atlas-based method that determines bone age by comparing the examiner's radiographs of the hands and wrists with the most similar standard radiographs in the GP atlas. The TW3 method, which has been modified twice, is a scoring system that measures individual bone maturity by scoring and summing multiple bones, such as metacarpal, phalanx, and carpal bones, and is a quantitative method. It is more accurate than the GP method but more time consuming [2, 3]. GP is the most popular method among pediatricians and radiologists, as BAA by GP is relatively quick and easy to learn. However, the GP method itself has significant inter- and intra-observer variability [4]. The TW method is considered to be more accurate and objective than the GP method and to have lower variability than GP [5, 6]. Will the variability be decreased further with the assist of AI-assisted diagnosis software?

With the rapid development of deep learning algorithms and the rapid improvement of computer hardware in the past few years, artificial intelligence AI-assisted diagnosis software has begun to be applied in hospitals, among which bone age AI-assisted software is one of the earliest [7–12]. AI-assisted diagnosis software for bone age has achieved good diagnostic performance [12–17]. Some studies have proven that the results of AI-assisted diagnosis software for bone age are as accurate as those of experts [13, 15, 18]. While some shown that AI assistance improves the diagnostic accuracy rate of radiologists [12, 15, 17, 19]. A few papers have focused on the interobserver agreement of radiologists, but the number of residents who participated seems inadequate[15, 20]. Little research has been performed on intra-observer variability (variation within individual observers) about the impact of AI-assisted software.

Herein, we evaluate an AI-assisted software designed to assist radiologists in the X-ray BAA interpretation. The purpose of this study was to investigate the effect of AI-assisted software on residents’ interobserver agreement and intraobserver reproducibility for the X-ray bone age assessment of preschool children.

## Methods

This study was approved by the Institutional Review Board and Ethics Committee of Peking University First Hospital, Beijing, China (IRB No. 2017–1382). Our study was exempt from the requirement of informed consent because of the retrospective nature of the study and the anonymous data.

## Patients

The studies were extracted and anonymized from 1320 left wrist X-ray images over a 1-year period from January 2018 to December 2018. Stratified random sampling by age and gender was performed from the children with a physiological age of 3–6 years old in preschool stage. For each age, 14 cases including 7 males and 7 females were included in the reading database. A total of 56 cases were included in the data set. Severe osteochondrodysplasia of the left wrist X-ray images were excluded form data set. The X-ray images with skeletal age exceeding the lower limit of the used standard were also excluded. None of the cases in this reading database participated in the training and verification of the AI software.

### AI-assisted software for bone age assessment

The bone age AI-assisted diagnosis software used in the study was provided by the Deep Wise Artificial Intelligence Lab which has got approval of National Medical Products Administration of China for clinical use. The development of the software follows the modified TW3 standard (modified for the Chinese people), which had been approved by the national official standards certification center and been widely used for BAA in China since 2006. The software is based on X-ray image preprocessing and a deep learning network for detecting and grading the wrist epiphysis to realize automatic identification and bone age assessment. For modified TW3-RUS, the mean absolute difference (MAD) was 0.25 years (95% confidence interval, 0.27–0.32 years) between the AI assessment and the reference standard. For modified TW3-carpal, the MAD was 0.17 years (95% confidence interval, 0.26–0.29 years) between the AI assessment and the reference standard.

### Study design and image interpretation

A crossover study design was used in the image interpretation process. A total of 6 board-certified residents were trained with the modified TW3 standard before bone X-ray interpretation. All residents underwent familiarization with the reading and reporting system before formal interpretation. The residents performed BAA independently in a reading room with a high-resolution monitor. The images were anonymized with all readers blinded to the clinical history and patient characteristics. The BAA assessment was carried out in PACS system by using structured report developed for modified TW3 standard BAA.

**Fig. 1:**
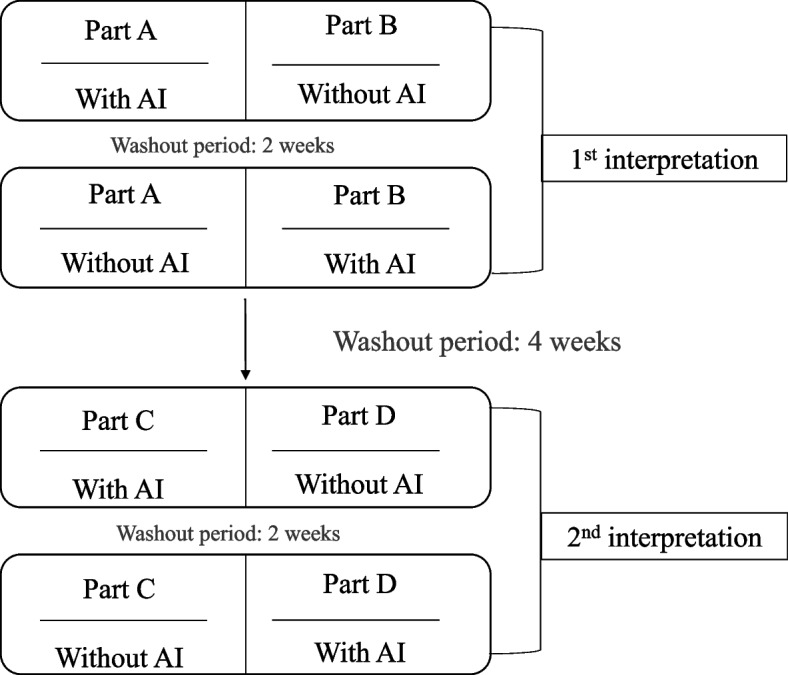
Flow chart of image interpretation for 6 residents. (The database was divided randomly and equally into A & B for the first interpretation and C & D for the second interpretation. Part A, B, C, D were not the same for each resident)

All the residents performed the image interpretation twice, with a 4-week washout period between the two interpretations. To reduce the influence of errors brought by memory, for each interpretation, a two-step random cross-reading method was used. The images in the database were randomly divided into two parts: one part was interpreted with AI assistance, and the other was interpreted without AI assistance, with a 2-week washout period between the two steps. All the BAA process included TW3-RUS and TW3-carpal. The crossover study design is shown in Fig. 1.

### Reference bone age

The reference bone age was determined by three pediatric radiologists with 12, 18, and 23 years of clinical experience who were familiar with bone age assessment based on X-ray radiographs. The average of the independent results of the three experts was used as the gold standard for this study. In case of a discrepancy over 2 years, the image would be discussed together until a consensus was reached.

### Statistical analyses

Statistical analysis was performed by using SPSS v19 (SPSS Inc., Chicago, Illinois, USA). For comparison of the accuracy of bone age between “without AI” and “with AI”, the root mean squared error (RMSE), mean absolute difference (MAD) and bone age accuracy within 0.5 years & 1 year of the 1st interpretation were used as metrics.

Interobserver agreement. For the 1st interpretation, the intraclass correlation coefficients (ICCs) with 95% confidence intervals for the 6 residents (residents 1–6) were compared between the results with and without AI. For the 2nd interpretation, the ICCs for the 6 residents (residents 1–6) were also compared in the same way. An ICC value greater than 0.75 is excellent, from 0.75 to 0.60 is good, from 0.59 to 0.40 is fair and below 0.40 is poor agreement[15, 18, 21].

Intraobserver reproducibility. Intraobserver agreement comparing the results of the same resident’s interpretations at two different times for all of the residents was determined via intraclass correlation coefficient (ICC) with 95% confidence intervals.

## Results

### Patients

Among the 56 cases, 2 cases were excluded due to severe osteochondrodysplasia, and 54 images were enrolled in the final database. Three cases in the "TW3-RUS" were excluded because the gold standard exceeded the lower limit of the modified TW3 standard. Fifty-one cases in the "TW3-RUS" were finally included in the final analysis. Eight cases in the "TW3-Carpal" were excluded because the gold standard exceeded the lower limit of the modified TW3 standard. Forty-six cases in the "TW3-Carpal" were finally included in the final analysis. The distribution of sex and age for all cases is presented in Table 1.

**Table 1:** Sex and chronological age distribution

	**Age**	**Male**	**female**	**Total**
**TW3-RUS**	3 years	5	5	10
	4 years	7	6	13
	5 years	7	7	14
	6 years	7	7	14
	Total	26	25	51
**TW3-Carpal**	3 years	7	1	8
	4 years	7	4	11
	5 years	7	6	13
	6 years	7	7	14
	Total	28	18	46

### Model accuracy in BAA

With the assistance of bone age AI software, the accuracy of residents’ results improved significantly. The average RMSE of TW3-RUS decreased from 0.806 years to 0.501 years, while the average MAD decreased from 0.608 years to 0.379 years. The accuracy increased from 56.4% to 69.6% within 0.5 years. The accuracy increased from 77.6% to 91.3% within 1 year. The TW3-RUS interpretation accuracy is presented in Table 2. The average RMSE of TW3-Carpal decreased from 0.508 years to 0.323 years, and the average MAD decreased from 0.355 years to 0.229 years. The accuracy increased from 67.4% to 82.6% within 0.5 years. The accuracy increased from 93.5% to 100% within 1 year. The TW3-Carpal interpretation accuracy is presented in Table 3.

**Table 2:** TW3-RUS interpretation accuracy in the 1st interpretation

	**average RMSE**	**average MAD**	**accuracy within 0.5 year**	**accuracy within 1 year**
without AI	0.806	0.608	56.4%	77.6%
with AI	0.501	0.379	69.6%	91.3%
Elevated value	0.305	0.229	13.10%	13.8%

**Table 3:** TW3-Carpal interpretation accuracy in the 1st interpretation

	**average RMSE**	**average MAD**	**accuracy within 0.5 year**	**accuracy within 1 year**
without AI	0.508	0.355	67.4%	93.5%
with AI	0.323	0.229	82.6%	100%
Elevated value	0.186	0.126	15.2%	6.5%

### Comparison of interobserver agreement

The results of interobserver agreement for diagnostic consistency are presented in Table 4. For the interobserver agreement comparison of TW3-RUS, the ICC results among 6 residents were elevated from 0.833 to 0.977 with the assistance of AI in the 1st interpretation and from 0.897 to 0.975 in the 2nd interpretation. For the interobserver agreement comparison of TW3-Carpal, the ICC results among 6 residents were elevated from 0.902 to 0.977 with the assistance of AI in the 1st interpretation and from 0.896 to 0.948 in the 2nd interpretation.

**Table 4:** Interobserver agreement of residents

	**TW3-RUS**		**TW3-Carpal**	
	without AI	with AI	without AI	with AI
	ICC (%95 CI (min–max))	ICC (%95 CI (min–max))	ICC (%95 CI (min–max))	ICC (%95 CI (min–max))
**1st interpretation**	0.833 (0.767–0.890)	0.977 (0.965–0.985)	0.902 (0.851–0.942)	0.977 (0.963–0.987)
**2nd interpretation**	0.897 (0.828–0.921)	0.975 (0.963–0.984)	0.896 (0.842–0.938)	0.948 (0.920–0.970)

### Comparison of intraobserver reproducibility

The results of intraobserver reproducibility are presented in Table 5. For intraobserver reproducibility of TW3-RUS between the 1st reading and 2nd reading, the ICC results with AI assistance were higher than the results without AI assistance for each resident. The results were similar for TW3-Carpal.

**Table 5:** Intraobserver reproducibility of residents

	**TW3-RUS**		**TW3-Carpal**	
	**without AI** between 1^st^ and 2^nd^ interpretation	**with AI** between 1^st^ and 2^nd^ interpretation	**without AI** between 1^st^ and 2^nd^ interpretation	**with AI** between 1^st^ and 2^nd^ interpretation
	ICC (%95 CI (min–max))	ICC (%95 CI (min–max))	ICC (%95 CI (min–max))	ICC (%95 CI (min–max))
**Resident 1**	0.793 (0.663–0.876)	0.986 (0.976–0.992)	0.888 (0.793–0.941)	0.976 (0.955–0.988)
**Resident 2**	0.870 (0.783–0.924)	0.971 (0.950–0.984)	0.930 (0.869–0.964)	0.975 (0.953–0.987)
**Resident 3**	0.898 (0.828–0.941)	0.959 (0.930–0.977)	0.860 (0.744–0.925)	0.891 (0.799–0.943)
**Resident 4**	0.857 (0.762–0.916)	0.986 (0.975–0.992)	0.906 (0.826–0.951)	0.977 (0.956–0.988)
**Resident 5**	0.951 (0.916–0.972)	0.991 (0.985–0.995)	0.936 (0.880–0.967)	0.969 (0.940–0.984)
**Resident 6**	0.802 (0.678–0.882)	0.976 (0.958–0.986)	0.922 (0.853–0.959)	0.967 (0.936–0.983)

## Discussion

X-ray bone age interpretation is widely used for growth and development assessment. Traditional methods are repetitive and time consuming. Deep learning (DL) could provide faster and more consistent interpretation. In this multi-reader study, changes in diagnostic accuracy, interobserver agreement and intraobserver reproducibility with and without AI assistance were investigated. The results showed with the assistance of bone age AI software, the diagnostic accuracy of bone age assessment can be improved for less experienced radiologists. Furthermore, AI-assisted software can eliminate both inter- and intra-rater variability.

With the use of AI and machine learning, especially the most well-known machine learning method deep learning, new possibilities for automated BAA have emerged[8–10]. The most popular deep learning is convolutional neural networks (CNNs), which have made tremendous progress in recent years, and there are numerous publications about the use of CNNs in BAA[15–18]. The Radiological Society of North America (RSNA) launched a BAA challenge in 2017, and many machine learning methods achieved good results[22]. The AI tool used in our study is also based on the CNN method. Skeletal maturity varies by ethnicity, geographic location, and socioeconomic status. Caucasian reference standards cannot be expected to be used for comparison in China. Therefore, the modified TW3 standard for Chinese people was applied in our research. The AI software used in the research was also developed based on the modified TW3 standard.

Environmental factors and endocrine diseases have different effects on RUS bone and carpal bone development[23–25]. In order to differentiation of bone development status and auxiliary diagnosis of diseases in children, RUS bone and carpal bone are assessed respectively since TW2 standard[26]. But AI-related research mainly focused on RUS bone. TW3-Carpal which is also important for BAA, was less evaluated than TW3-RUS. In our study, we designed our study to investigate the variability both for TW3-RUS and for TW3-Carpal. This is also one of the advantages of this study.

The emergence of fully automatic AI software helps us overcome complexity and time consumption in the interpretation process. Most publications discuss the data between AI and radiologists with convincing good results about improved accuracy or reduced complexity and time. However, it is not yet the reality to send the AI results directly to the pediatrician without confirmation by a radiologist. In clinical practice, the purpose of AI-assisted software is to assist the radiologist but not to use it independently. Only by validating the results of AI-assisted software in daily routine can it truly prove its value. Therefore, two image interpretation scenarios with and without AI were included in our research.

One of the challenges in BAA is the variability in radiologist clinical interpretation of bone age radiographs, both for inter- and intra- observer. Will automated bone age tools eliminate enhanced interobserver diagnostic consistency or intraobserver diagnostic reproducibility? Tajmir et al.[20] revealed that BAA with DL improved the radiologist performance while decreasing the variation (ICC without AI was 0.9914, with AI was 0.9951). But only three radiologists participated in image interpretation. Lee et al.[15] developed a deep learning-based hybrid (GP and modified TW) method for BAA, and the ICC of the two radiologists slightly increased with AI model assistance (from 0.945 to 0.990). In another study by Koc et al.[18], the ICC was 0.980 without AI and 0.980 with AI (BoneXpert). The interobserver variability was not eliminated in their research. In our study, for the interobserver agreement comparison, the ICC results among 6 residents were elevated up to 0.977 for both TW3-RUS and TW3-Carpal. For intra-observer reproducibility between the 1st reading and 2nd reading, the ICC results were elevated up to 0.991 (resident 5) for TW3-RUS and up to 0.977 (resident 4) for TW3-Carpal. AI bone age tools can eliminate both interobserver variability and intraobserver variability.

Our study has limitations. First, this was a single-center study with a small and single-ethnicity sample size, and only preschool children were enrolled. In the future, prospective multicenter studies with more cases will be performed. Second, the interpretation time was not recorded. The time consumption should be compared, although many studies have already demonstrated that AI-assisted software can obviously reduce the diagnostic time [3, 6, 12].

For preschool children X-ray bone age assessment, in addition to improving diagnostic accuracy, bone age AI-assisted software can also increase interobserver agreement and intraobserver reproducibility. AI-assisted software can be an effective diagnostic tool for residents during BAA.

## Data Availability

The datasets used and/or analysed during the current study are available from the corresponding author on reasonable request.

## Abbreviations

AI: Artificial intelligence
BAA: Bone age assessment
GP: Greulich-Pyle
ICC: Intraclass correlation coefficient
MAD: Mean absolute difference
PACS: Picture archiving and communication system
RMSE: Root mean squared error
TW3: Tanner-Whitehouse 3
